# UV irradiation behavior of *Salix psammophila* sand barriers in the process of desertification control

**DOI:** 10.3389/fpls.2024.1451065

**Published:** 2024-09-13

**Authors:** Ruidong Wang, Shichao Chen, Yong Gao

**Affiliations:** College of Desert Control Science and Engineering, Inner Mongolia Agricultural University, Hohhot, China

**Keywords:** desertification control, *Salix psammophila* sand barriers, performance degradation, ultraviolet irradiation, biological resources utilization

## Abstract

*Salix psammophila* sand barriers degrade under sunlight exposure, resulting in diminished protective performance and shortened service life in desertification control. To address the unresolved issue of photoinduced damage and degradation in sand barriers, we conducted simulations to assess the accelerated damage effect of ultraviolet (UV) rays during solar exposure of *S. psammophila* sand barriers. Our analysis focused on elucidating the mechanism of UV irradiation in sand barriers by examining the structural and material property changes that occur during the degradation process. The results indicated the following: (1) The discoloration of sand barriers resulting from UV irradiation was primarily ascribed to the modification in lignin content. (2) The morphology and protective performance of *S. psammophila* sand barriers underwent significant changes following exposure to UV irradiation. The 96-day and 144-day time points of UV exposure are crucial for evaluating the extent of UV degradation in sand barriers. After 192 days of UV irradiation, there was a decrease in mass loss percentage by 3.62%, modulus of elasticity by 8.63%, and modulus of rupture by 6.74%. (3) The lignin, hemicellulose, and cellulose content decreased by 23.12%, 14.30%, and 6.96%, respectively. The impact of UV irradiation on the polysaccharide (cellulose and hemicellulose) in *S. psammophila* sand barriers was relatively minimal. (4) The carbon binding form in *S. psammophila* sand barriers underwent a transformation, characterized by a significant decrease in C1 content and an increase in C2 and C3 content. This resulted in a gradual enhancement of the oxidation state and binding energy of carbon. Therefore, to prolong the utilization lifespan of *S. psammophila* sand barriers, it is essential to address the UV irradiation behavior from the perspective of inhibiting lignin reactions.

## 1 Introduction

As one of the numerous global environmental issues in the contemporary world, desertification has threatened the survival and development of human society, restricted the sustainable development of the social economy, and drawn increasing attention from desertification control workers and researchers worldwide (Li et al., 2018). In recent years, relevant researchers have developed diverse sand damage prevention and control technologies applicable to specific environments, which have, to a certain extent, provided reference experiences for the large-scale application of desert management models (You et al., 2021; Cui et al., 2023). *Salix psammophila* (C. Wang and C. Y. Yang) is a perennial shrub species that is widely distributed in arid and semi-arid regions. It possesses the traits of cold tolerance, drought resistance, high-temperature tolerance, sand burial resistance, and strong adaptability. It can thrive extensively in environments with adverse wind and sand conditions, drought, and scarce rainfall, and is an ideal sand control material in Northwest China and Western Inner Mongolia (Gao et al., 2013). Many scientists employ fresh *S. psammophila* strips as materials to establish various forms of sand barrier configuration patterns on the sand surface, thereby controlling the direction, speed, and structure of the wind–sand flow and altering the situation of wind erosion and sand accumulation on the dune surface. This has become one of the crucial sand control methods for scientific and efficient resource utilization in the local area (Gao et al., 2013). Gao and Yu (1996) demonstrated that setting *S. psammophila* sand barriers on mobile dunes can effectively enhance the dune microenvironment and facilitate and accelerate the process of vegetation restoration Gao and Yu, 1996). Simultaneously, some scholars discovered that *S. psammophila* sand barriers had a markedly positive effect on increasing vegetation coverage (Ren et al., 2007; Zhao et al., 2008).

*S. psammophila* wicker, as a natural wood material, is cut down and made into *S. psammophila* sand barriers after interception. Long-term exposure to the atmospheric desert environment and the elements (sunlight, oxygen, temperature, and chemical media) inevitably affects them, resulting in cracking, warping deformation, and lodging (Zhou et al., 2008). This can cause irreversible changes in the physical and mechanical properties of the sand barriers, resulting in the gradual loss of their excellent protective capability, thereby affecting the wind and sand prevention effect of the sand barriers (Wang et al., 2019; Yang et al., 2021). Among these factors, the light energy of solar radiation is the most detrimental factor to plant wood materials in the outdoor environment and, to a certain extent, determines the service life of sand barrier materials. In the process of actual use in the field of desertification control, the protection benefit of *S. psammophila* sand barriers can only last for 5 to 7 years (Ren et al., 2007; Gong, 2012). Currently, the problem of ultraviolet (UV) irradiation has become one of the main factors restricting the application of sand barriers (Wang et al., 2021). A study on the effect of UV irradiation on the performance of sand barriers has not been carried out, and the research on the aging mechanism is lacking. This knowledge gap greatly limits our understanding of the deterioration process of sand barriers caused by UV irradiation in the desert-exposed atmospheric environment. In the early stage, the research group preliminarily explored the changes of the structure and performance of the sand barriers and found that key environmental factors such as UV irradiation in the exposed atmospheric environment had an impact on the sand barriers, but it did not clarify how UV irradiation affected the structure and performance degradation of the sand barriers and how the mechanism acted, especially in the harsh desert environment. Hence, it is essential to further elucidate the alterations in the structure and properties of sand barriers throughout the aging process.

Owing to the prolonged and uncontrollable characteristic of UV irradiation on wood materials exposed to sunlight, our research centered on theoretically investigating the photoaging of sand barriers utilized in Inner Mongolia under harsh geographical and climatic circumstances with high UV radiation intensity. To precisely simulate the influences of UV irradiation on sand barriers, we adopted an indoor artificial strong UV irradiation source ring box test approach to replicate the exposure of sand barriers to sunlight. This method enabled us to convert outdoor UV irradiation time into equivalent indoor strong UV irradiation time, thereby shortening the simulation aging degradation period and effectively simulating the impact of sunlight exposure on the performance of *S. psammophila* sand barriers. Our study comprehensively and systematically depicted changes in the composition of sand barriers during UV irradiation, and proposed efficient ways for enhancing their light resistance. These discoveries aim to offer valuable perspectives for related research undertakings.

## 2 Materials and methods

### 2.1 *Salix psammophila* sand barrier sample selection and experimental design

Experimental materials: The materials were collected from Hangjin Banner, Ordos, Inner Mongolia. The representative 3- to 5-year mature and healthy growing fresh *S. psammophila* branches were randomly selected in the collection area. *S. psammophila* branches with a diameter class of 7–13 mm were selected 20 cm from the ground as experimental materials. According to the national forestry industry standard LY/T 2369-2014 “Testing Method for Physical and Mechanical Properties of Desert Shrubs”, sawing and cutting were carried out (LY/T 2369, 2014). When selecting specimens, defective specimens formed in the sawing process, with a very rough surface and uneven thickness, and no large numbers or obvious joints, cracks, and other defects were first removed. The purpose is to avoid the difference in results caused by the different physical, mechanical, and chemical properties and structures of *S. psammophila* at different locations and to minimize the discreteness of the test results.

### 2.2 Design of the indoor UV irradiation simulation experiment

An Artificial Intensive Ultraviolet Radiation Environment Box (AIUREB) was used for the artificial accelerated UV irradiation simulation experiment. According to the standard ASTM154-06 (Standard Practice for Operating Fluorescent Light Apparatus for Exposure of Nonmetallic Materials) test method for accelerated aging experiment, the total effective time of outdoor UV radiation is converted into indoor simulation time of UV radiation. The reference standard of outdoor UV radiation quantity is the natural condition of the Kubuqi desert area in Inner Mongolia. The average total solar UV radiation in the Inner Mongolia Autonomous Region from 1961 to 2017 was 4,860–6,931 MJ/m^2^, and solar energy resources were very rich. Affected by geographical location and climate factors, sunshine duration was relatively long. The annual total UV radiation in the study area was 6,581.2 MJ/m^2^ (658.12 kJ/cm^2^). Among them, the total UV radiation accounts for 7% of the total solar radiation, that is, 46.07 kJ/cm^2^. Based on the irradiance meter values, the UV irradiation intensity is the strongest at a distance of 30 cm from the light source, and the average UV irradiation intensity is 0.024 W/cm^2^, according to the conversion method of the total effective time of outdoor UV radiation and the total effective time of indoor UV radiation (Qi, 2011; Yan et al., 2001).

According to the calculation of the pre-test UV radiation amount and the limitation of the test conditions, the maximum degree of light aging damage of the outdoor natural exposure of the *S. psammophila* sand barriers is simulated. During the experiment, the daily exposure time is controlled from 6:30 a.m. to 10:30 p.m. every day. The total UV irradiation is 16 h, with an interval of 8 h, to simulate the phenomenon of day and night alternating. Intermittent UV irradiation helps crack the test material of *S. psammophila* sand barriers, so that its inner structure can receive UV radiation faster (Ye and Huang, 2005). The natural UV radiation time, laboratory simulation time, and radiation amount in the Kubuqi Desert area can be converted by the formula, as shown in **Table 1**.

**Table 1:** UV aging simulation time and radiation amount of *S. psammophila* sand barrier.

Outdoor UV irradiation time (years)	Total outdoor UV irradiation (kJ/cm^2^)	Indoor UV irradiation time (days)	Total indoor irradiation (kJ/cm^2^)
0	0	0	0
1	46.07	24	50.68
2	92.14	48	101.35
3	138.21	72	152.03
4	184.28	96	202.71
5	230.35	120	253.39
6	276.42	144	304.06
7	322.49	168	354.74
8	368.56	192	405.42

During the test, the temperature of the environment chamber during the test was controlled to 40 ± 3°C to prevent the sample from thermal aging due to the excessive temperature during UV aging, which would affect the accuracy of the test. After the test, the test samples were taken out respectively to test the performance index of the *S. psammophila* sand barriers. According to the sampling aging time point, the samples were divided into eight time cycles; each cycle had 1 to 10 parallel replicates, for a total of 344 test materials. After the end of the optical aging test, the aging sample of each different UV treatment period is placed in an artificial climate chamber with a temperature of 20 ± 0.2°C and a relative humidity of 65% ± 5%, and the moisture content of the sample is adjusted to achieve the moisture content of 9%–15% required by the national standard mechanical test. Physical and mechanical property parameters were measured respectively. After the index test was completed, some samples were air-dried and ground into wood powder. The main chemical composition, functional group, and chemical bond changes of each type of sample were further tested, so as to explore the damage mechanism of mechanical properties of sand barrier caused by light aging from a chemical perspective. At the same time, some samples were selected to make slices, and the change of sample structure after photoaging was observed.

### 2.3 Experimental test method

#### 2.3.1 Observation of the structure of *Salix psammophila* sand barriers after ultraviolet light aging

Macroscopic structure observation: The samples retrieved from the field and the *S. psammophila* sand barrier samples treated in different rooms were polished, and the blocks were repaired with knives and other tools. Some samples were selected and observed with a stereomedical microscope (Leica SAPO, Leica, Switzerland).

Microstructure observation: The parts of the sand barrier exposed to the atmosphere are selected in the field test, and some samples with serious changes and differences are selected in the indoor simulation test for microstructure observation. Horizontal and radial test blocks were prepared according to the national standard GB/T29894, 2013. The sample material was vented through a water bath for 10 days. Glacial acetic acid and 30% hydrogen peroxide were boiled and softened for 30 minutes at a ratio of 2:1. The acid solution was removed by rinsing several times with distilled water and finally soaked in ether solution for testing. Slides with a thickness of approximately 10–20 μm were prepared using a slip-on microtome (SM2400, Lycra, Germany), and then observed with a conventional light microscope and a scanning electron microscope.

#### 2.3.2 Color difference test of *Salix psammophila* sand barriers

Colorimetric parameters of UV irradiation sand barriers were measured by a colorimeter (CM-2300D, Konica Minolta, INC, Japan). Colorimetric parameters were measured at nine points on each sample, and the results were averaged. Colorimetric parameters ΔL*, Δa*, and Δb* refer to the CIE1976 L*a*b* system to determine the total color difference (ΔE*) that represents the corresponding difference between the optical aging test material and the control group, and the calculation formula is as follows:


$$\text{Δ}{\text{E}}^{*}=\;\left(\text{Δ}{\text{L}}^{*}2+\text{Δ}{\text{a}}^{*}2+\text{Δ}{\text{b}}^{*}2\right)\;1/2$$

In the formula, ΔL*, Δa*, and Δb* respectively represent the difference of brightness value, red-green value, and yellow-blue value before and after aging of the test material.

#### 2.3.3 Physical and mechanical property test of *Salix psammophila* sand barriers

The section with an intercept length of 70 ± 0.2 mm of the sample is placed in a constant temperature and humidity box with a temperature of 20 ± 2°C and a relative humidity of 65% ± 5%. The moisture content of the *S. psammophila* sand barrier sample is adjusted for it to reach the 9%–15% moisture content required by the national standard mechanical test for physical and mechanical index testing (Raczkowski, 1980). Physical properties such as weight loss percentage, basic density, dry shrinkage, and modulus of elasticity decreased by 8.63%, and the modulus of rupture properties were determined and calculated according to national forestry industry standard LY/T 2369-2014 “Testing Methods for Physical and Mechanical Properties of Desert Shrubs”. The test equipment is the electronic universal testing machine (TY8000 type) of Jiangsu (China) Tianyuan Test Equipment Co., Ltd.

#### 2.3.4 Chemical component analysis of *Salix psammophila* sand barriers

Part of the *S. psammophila* sand barrier samples were selected for the determination of the main chemical components in the field exposed to the atmosphere and different treatment simulation tests in the room. Part of the samples were selected for air drying under the full dry state and then ground into a powder form over a 40–60 mesh screen, and dried in an oven at 80°C, so as to more accurately weigh the constant weight of the samples. The cellulose content of *S. psammophila* sand barriers was determined by the nitro-ethanol method, the hemicellulose content was determined by the hydrochloric acid hydrolysis method, and the lignin content was determined by the Kalson lignin method (Wang and Cheng, 2011; Zhang et al., 2016; Xiong et al., 2005). The method has the advantages of high precision, good reproducibility, and easy to master.

#### 2.3.5 FTIR test analysis of *Salix psammophila* sand barriers

FTIR analysis of *S. psammophila* sand barrier samples was performed by using the traditional KBr tablet fabrication method. Wood powder sample (2 mg) and potassium bromide (200 mg) from *S. psammophila* sand barriers samples were mixed evenly and placed in the production mold, and round thin samples were made under 8–10 MPa for 2 min. A BRUKER (TENSON 27) infrared spectrometer was used for the experiment. The spectrometer is equipped with a DTGS detector, and the infrared spectrum scanning range is 4000–400 cm^−1^, the resolution is 4 cm^−1^, and the scanning times are 32. The OPUS 6.5 analysis software was used to conduct pretreatment such as baseline correction, atmosphere compensation, smoothing, and normalization (Traoré et al., 2016; Boukir et al., 2019), respectively, to determine the relative intensity of characteristic peaks of main chemical components of *S. psammophila* sand barriers.

#### 2.3.6 Analysis of x-ray diffraction

X-ray diffraction analysis of sand barrier samples was carried out by an x-ray diffractometer (Philips X-Pert, Panalytical, Almelo, Netherlands) under Cu-Kα radiation (*k* = 0.154 nm). The test conditions were as follows: 2 s/step, 0.02°C/step, 40 kV, and 30 mA, and the sample was spread over a range of 2θ = 5°C to 40°C. The Cri (%) crystallinity index was described by the Segal method (Segal et al., 1959), using the following equation (Boukir et al., 2019):


(1) $$C\text{r}.I(\%)=\left[\left(I_{002}-I_{\text{am}}\right)/{\text{I}}_{002}\right]\times 100$$

where I002 is the diffraction peak intensity of the crystallization corresponding to the (002) plane at 2θ = 22.5°C, and Iam is the diffraction intensity at 2θ = 18°C.

#### 2.3.7 X-ray photoelectron spectroscopy

XPS analysis was performed using x-ray photoelectron spectroscopy (Escalab 250Xi; Thermo Fisher Scientific, Waltham, Massachusetts, USA). In this study, *S. psammophila* sand barriers with different aging times (0, 5, and 7 years) were selected, and small samples with dimensions of 10 × 10 × 2 mm (length × width × thickness) were cut. An aluminum target x-ray source with a monochromator (Al Kα, Hν = 148,671 eV) was used at 225 W (operating voltage 15 kV, emission current 15 mA). Furthermore, the pollution carbon (internal standard) was 284.8 eV, the minimum energy resolution was 0.48 eV Ag (3d5/2), and the minimum XPS analysis area was 15 μm. The data were processed using MDI.Jade 6.0 and Origin 2021 software.

## 3 Results

### 3.1 UV irradiation phenomenon and color change of *Salix psammophila* sand barriers

**Figure 1:**
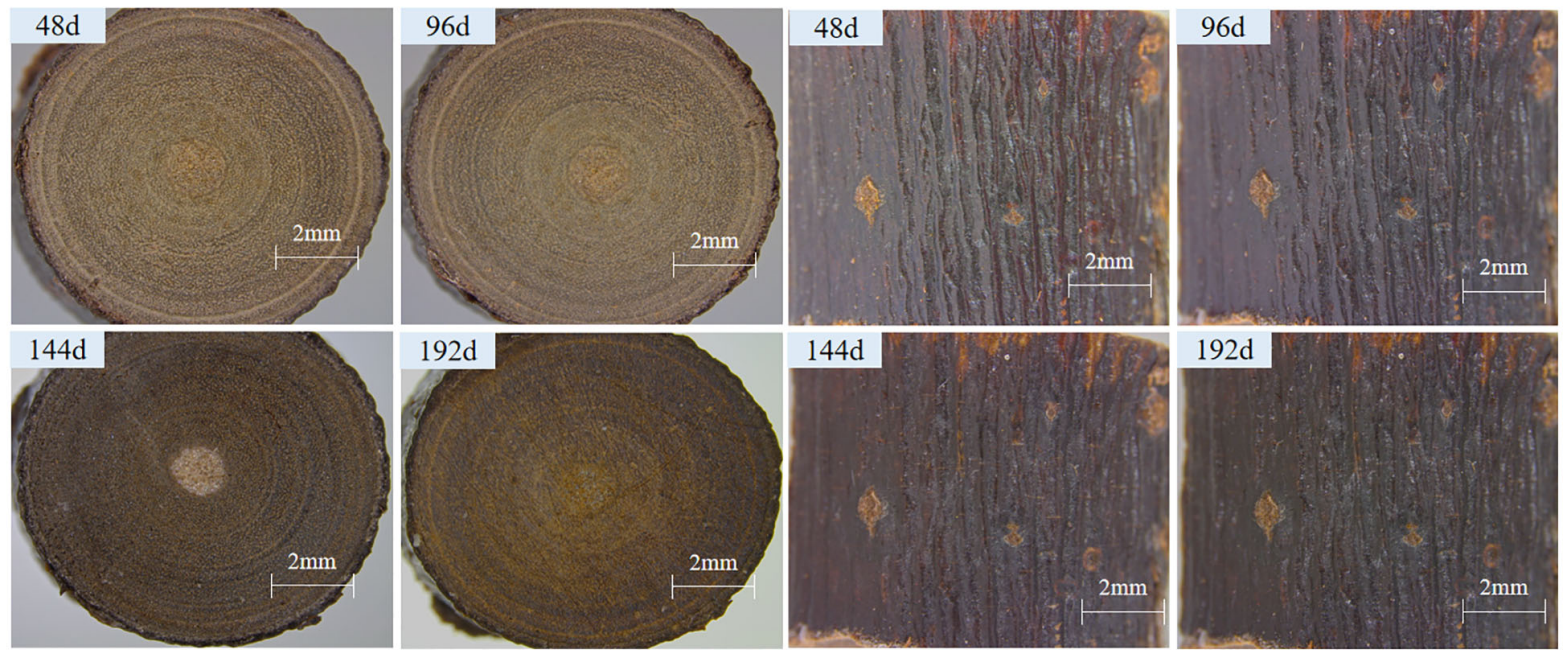
UV irradiation aging process cross-section and vertical section changes of *S. psammophila* sand barrier.

**Figure 2:**
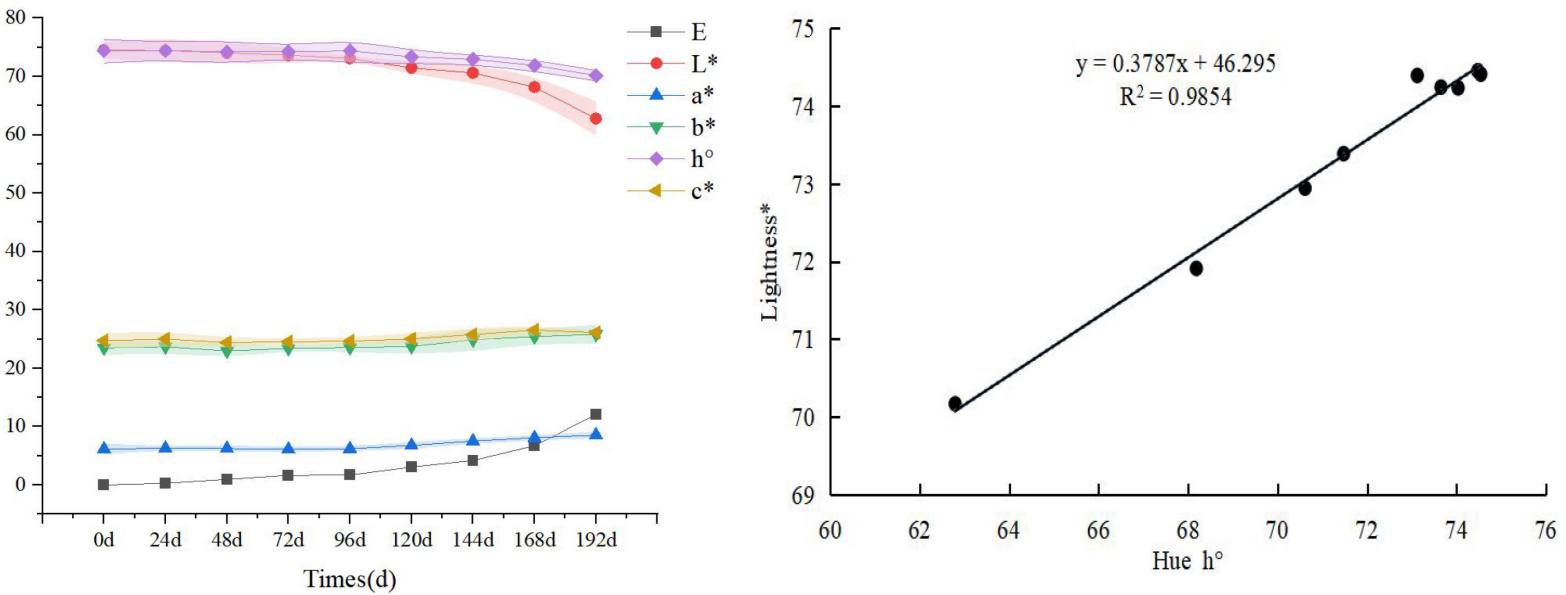
UV irradiation aging process cross-section and vertical section changes of *S. psammophila* sand barrier.

The changes of cross-section and longitudinal section of sand barrier samples during UV irradiation are shown in **Figure 1**, and color changes are shown in **Figure 2**. The appearance of *S. psammophila* sand barriers changes obviously after UV aging in different periods. In the process of UV irradiation from 0 to 96 days, the total color difference ΔE and lightness (L) of the sand barrier changed little, and the red-green axis chroma index a* and yellow-blue axis chroma index b* decreased slightly, indicating that the UV irradiation treatment had little effect on the color of the sand barrier during this period. After 96 days, with the increase of the UV irradiation period, obvious color changes have appeared on the surface, the lightness and gloss have decreased, there is a yellowish trend, and the color of the sand barrier itself has been lost. With the extension of UV irradiation time, the color changes from light brown to dark. After 120 days of UV radiation, the gloss basically disappeared, and the color change of the longitudinal section of the sand barriers was obvious at this stage. From the sixth cycle of UV irradiation (144 days), with the increase of the UV irradiation period, the yellow-blue coordinates began to decline, and there was a blue trend. The maximum irradiation time of a* and b* values increased, that is, reddening and yellowing were produced, with yellowing being more obvious, the overall change difference was small, and the change tended to become stable

The change trend of L and h values is the same, and they gradually decrease with the extension of UV irradiation time, while the values of a*, b*, and C gradually increase, and the values of L and h decrease at the same time, which makes the sand barrier much darker with the UV irradiation time. Because of the large number of parameters of the color system, considering the direct relationship between hue and red-green index a* and yellow-blue index b*, the correlation analysis between hue and lightness is carried out, and it is found that there is a good linear relationship between lightness and hue. Therefore, lightness can be used as a parameter to measure the color change of the UV irradiation of the sand barriers. UV irradiation processes color changes, indicating that UV irradiation chemical reactions have begun to occur on the surface.

### 3.2 UV irradiation on the physical and mechanical properties of *S. psammophila* sand barriers

#### 3.2.1 Physical properties of *Salix psammophila* sand barriers under UV irradiation

**Figure 3:**
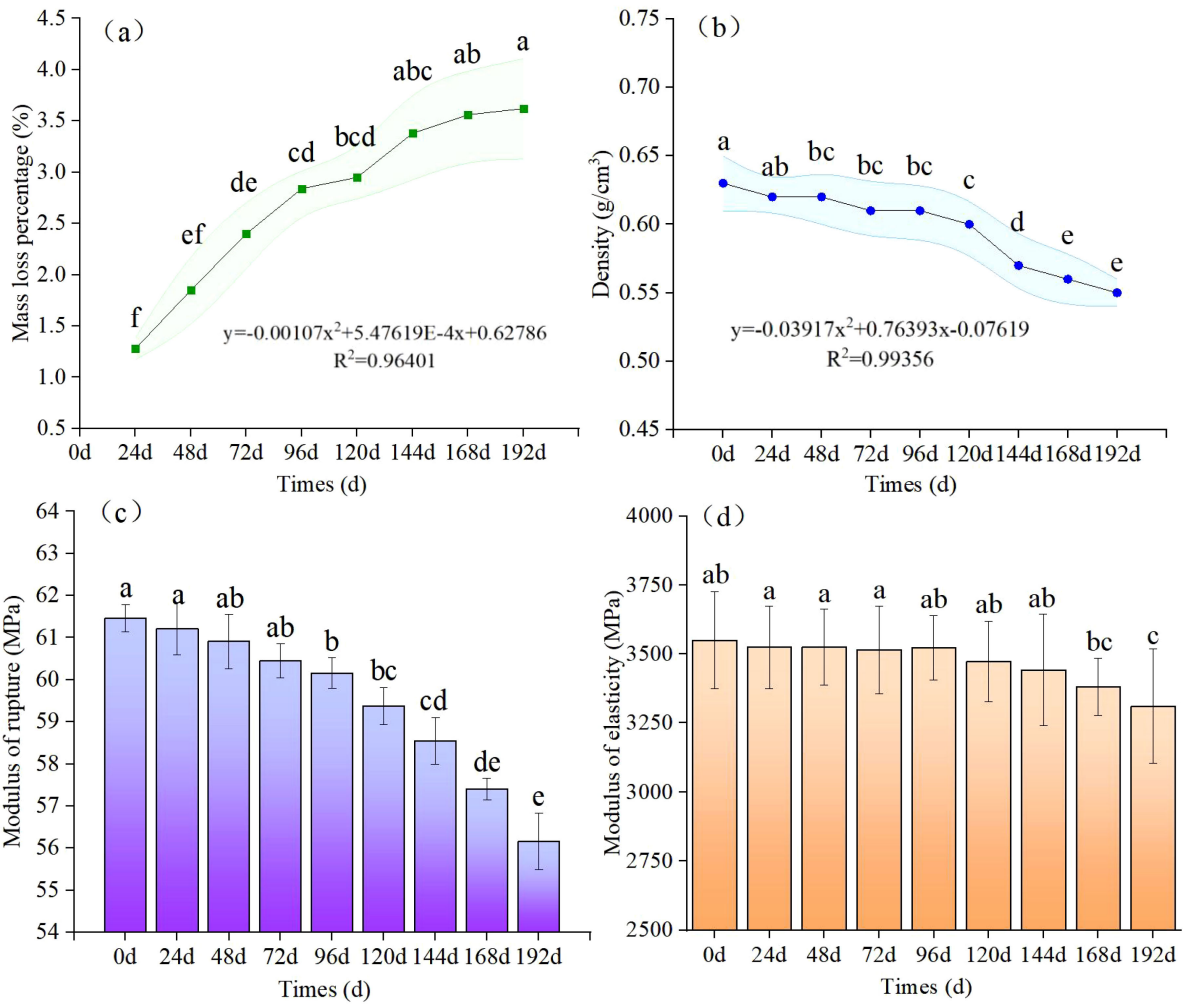
UV irradiation aging mass loss percentage, MOR, and MOE changes of *S. psammophila* sand barrier. Different lowercase letters in the figure indicate significant differences in different photoaging treatment weeks (*p* < 0.05). (**A** Mass loss percentage; **B** Density; **C** Modulus of rupture; **D** Modulus of elasticity).

The change trends of mass loss percentage and density of sand barriers under different UV irradiation conditions are shown in **Figure 3**. With the increase of UV irradiation time, the quality of sand barriers has been lost to different degrees, showing an overall increasing trend and a decreasing trend of density. After 192 days of UV irradiation, the mass loss percentage is only 3.62%, and the density of sand barriers decreases from 0.63 to 0.55 g/cm^3^. There was a strong linear relationship between mass loss percentage and density of sand barriers under different UV irradiation conditions (*p* < 0.05), *R* ^2^ = 0.94 and *R* ^2^ = 0.91. There were remarkable differences in UV irradiation treatment after 96 days, 144 days, and 192 days (*p* < 0.05). The results show that optical aging has little effect on the mass loss percentage and density of the sand barriers.

As can be seen from **Figure 3**, both the modulus of elasticity and modulus of rupture of the sand barriers show a downward trend during UV irradiation. After 192 days of UV radiation, the modulus of elasticity decreased by 8.63% and the modulus of rupture decreased by 6.74%. The influence of UV irradiation on the modulus of elasticity was slightly greater. During the process of UV irradiation from 96 to 192 days, the modulus of elasticity decreased remarkably, and the modulus of elasticity decreased from 60.26 to 56.16 MPa from 96 to 192 days, a decrease of 6.81%. While the modulus of rupture is accompanied by the decrease of modulus of elasticity, the regularity of decline is poor, but overall, it continues to show a downward trend with the UV irradiation, showing a slight downward trend before 96 days, and a rapid downward trend after 96 days.

At the same time, the physical and mechanical properties of sand barriers by UV irradiation were analyzed by one-way ANOVA. The physical and mechanical property indexes of sand barriers by UV irradiation were significantly different, and the influence on modulus of rupture was less different. The results showed that the physical and mechanical properties of sand barriers changed with the UV irradiation time, and there was a certain coordination relationship (*p* < 0.05). There are significant differences between 96 days and 0 days, 144 days, and 192 days, indicating that UV irradiation for 96 days is an obvious time inflection point (*p* < 0.05).

#### 3.2.2 Changes of main chemical composition of *Salix psammophila* sand barriers under UV irradiation

**Figure 4:**
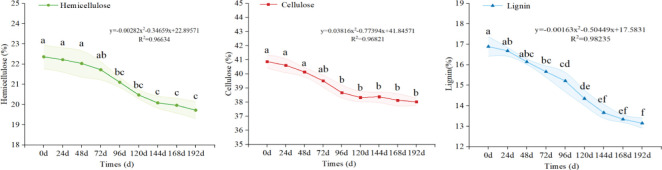
UV irradiation aging process and main chemical composition changes of *S. psammophila* sand barriers. Different lowercase letters in the figure indicate significant differences in different photoaging treatment weeks (*p* < 0.05).

As is shown in **Figure 4**, the content of cellulose, hemicellulose, and lignin in the *S. psammophila* sand barriers decreased with the increase of UV irradiation. After 96 days of UV radiation, the lignin content of the *Salix* sand barrier decreased by 11.08% from 17.11% to 15.21%. The hemicellulose content decreased by 8.30% from 23.01% to 21.10%. After 192 days of UV irradiation, the lignin content decreased from 15.21% to 13.15%, with a decrease of 13.54%. The hemicellulose content decreased by 6.54% from 20.20% to 16.13%. The cellulose content decreased by 1.66% from 38.67% to 38.02%. Throughout the entire 0- to 192-day UV irradiation cycle, there was a reduction in lignin, hemicellulose, and cellulose content by 23.12%, 14.30%, and 6.96% respectively, indicating that lignin is the primary chemical component affected by UV irradiation, with only a minimal amount of cellulose being decomposed. The contents of the main chemical components varied significantly across different UV irradiation periods (*p* < 0.05). 

### 3.3 Changes of microstructure and cellulose crystallinity of *Salix psammophila* sand barriers under UV irradiation

**Figure 5:**
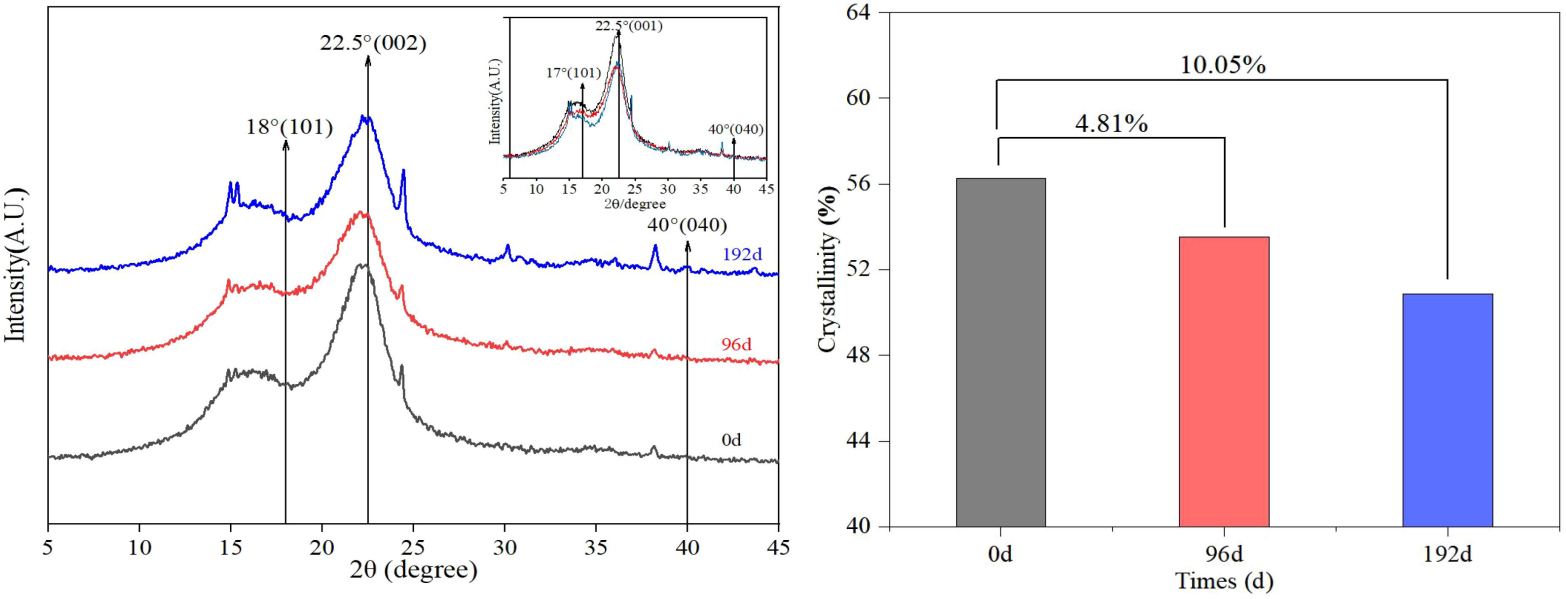
UV irradiation aging process and XRD and crystallinity content characteristic changes of *S. psammophila* sand barriers.

The maximum diffraction value of the sand barriers, radiated by UV irradiation, is 2θ = 22.5° (002) plane (**Figure 5**). The maximum scattering intensity in the amorphous region occurs near 2θ = 18° with no significant change observed and no new fiber beam diffraction peak generated, indicating that the lattice structure of cellulose remains undamaged after UV irradiation. **Figure 5** illustrates a slight decrease in the crystallinity of *S. psammophila* sand barrier with increasing UV irradiation time. After 96 and 192 days of UV irradiation, the crystallinity of cellulose decreases by 4.81% and 10.05%, respectively. This result may be due to the fact that the *S. psammophila* sand barrier, as a wood material, mainly degrades lignin in the process of UV irradiation and also degrades cellulose and hemicellulose to a certain extent, but the effect is weak.

### 3.4 Changes of chemical functional groups of *Salix psammophila* sand barriers under UV irradiation

**Figure 6:**
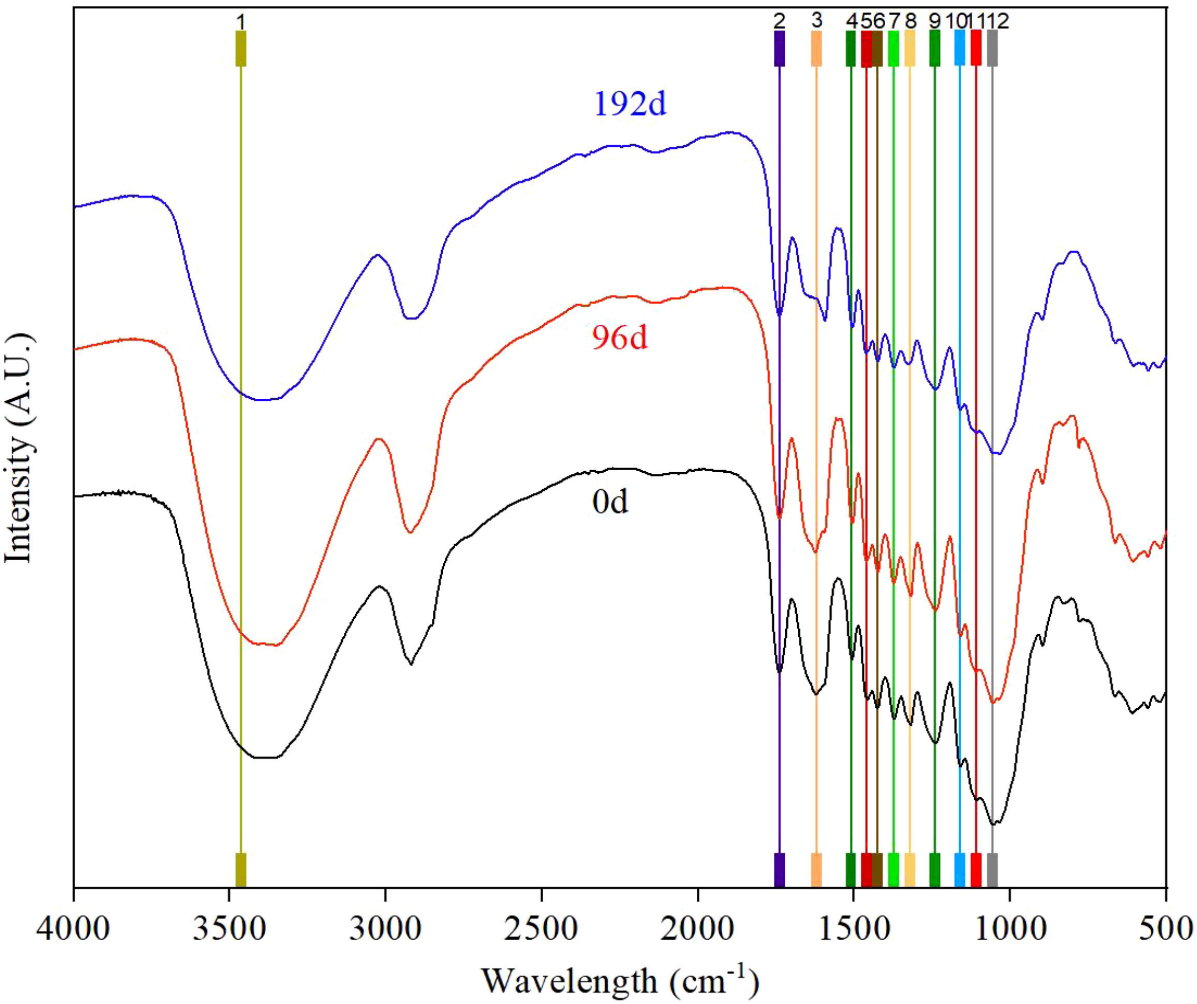
UV irradiation aging process and infrared spectra of *S. psammophila* sand barriers.

The FTIR spectral information of UV irradiation *S. psammophila* sand barrier samples after 96 and 192 days is shown in **Figure 6**. The characteristic absorption peaks of the sand barrier samples in the range of 3,500–1,000 cm^−1^ wave number are as follows. After 192 days of UV irradiation, the C=C group absorption peak is at 1,610 cm^−1^, the aromatic benzene ring skeleton vibration is at 1,510 cm^−1^, C-H in lignin and carbohydrates, 1425cm^−1^ (C-H in lignin and hemicellulose) and 1242cm^−1^ (Lignin C-O). The characteristic peaks changed obviously, indicating that the lignin chemical composition of *S. psammophila* sand barriers had UV irradiation. Among them, the deformation and vibration of the cellulose and hemicellulose C-H group at 1,373 cm^−1^, the association absorption band at 1,161 cm^−1^, and the stretching and stretching vibration frequency peak of the C-O group at 1058 cm^−1^, which characterized cellulose and hemicellulose, did not decrease obviously after UV irradiation, and UV irradiation did not induce significant degradation of polysaccharide. The change of the chemical groups representing lignin was smaller than that of lignin, indicating that cellulose and hemicellulose were less affected by UV radiation than lignin during UV irradiation.

### 3.5 X-ray photoelectron spectroscopy of *Salix psammophila* sand barriers under UV irradiation

**Figure 7:**
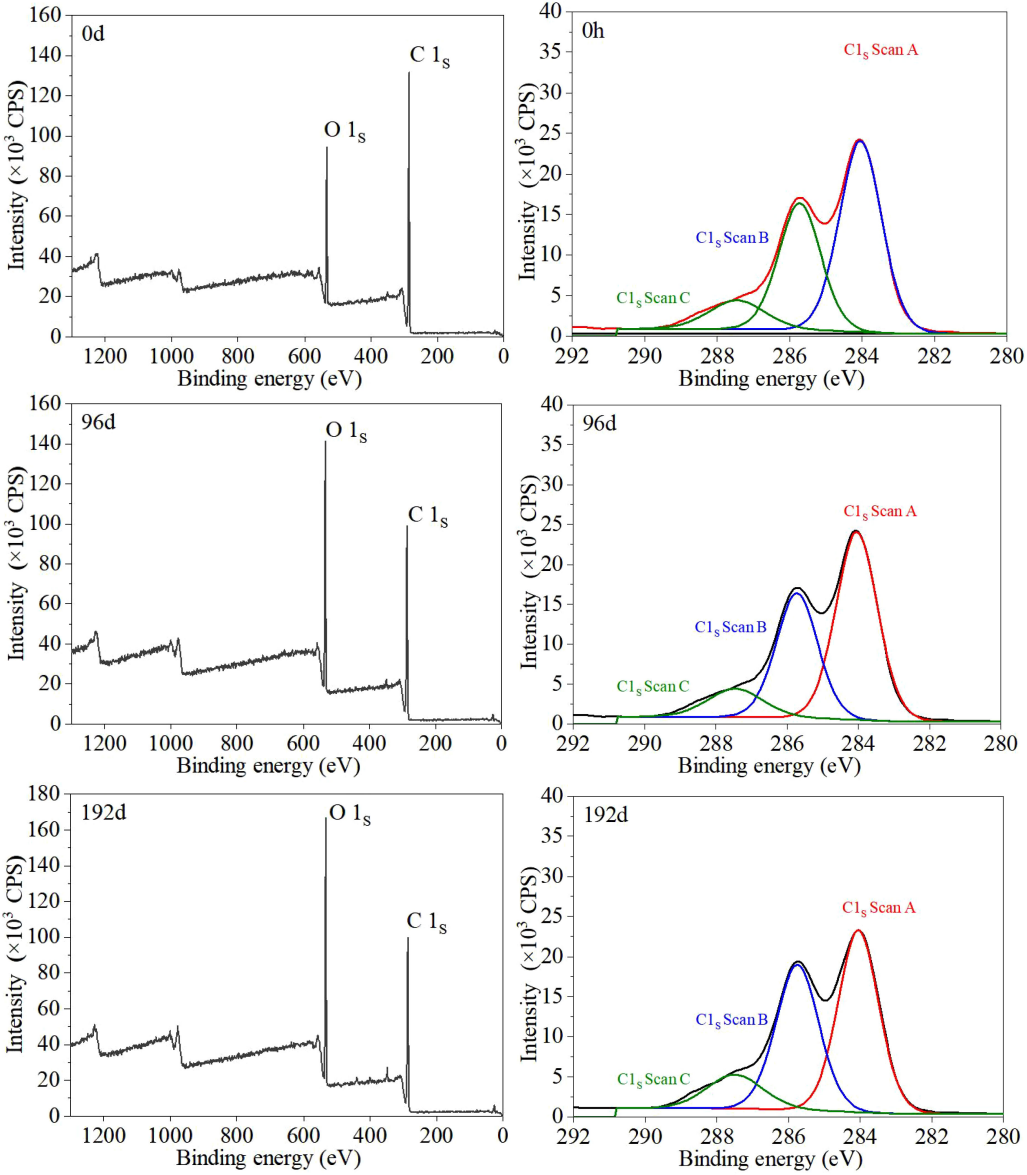
UV irradiation aging process, XPS survey spectra, and C1s narrow scan of *S. psammophila* sand barriers.

As shown in **Figure 7**, the XPS wide scanning spectrum information of the sand barriers irradiated by UV irradiation rays shows two obvious peaks, a C1s peak with an electron binding energy of approximately 285 eV and an O1s peak with an electron binding energy of 532 eV, in each spectrum. After 192 days of UV irradiation, the main chemical elements of the *S. psammophila* sand barriers are still C and O, but the relative content ratio of C and O elements changes. In order to further explore the mechanism of UV irradiation, we have carried out a detailed analysis of the atlas. According to the relative positions of binding energies of C1, C2, C3, and C4 of the standard spectrum, the C1S spectrum of the UV irradiation sand barrier samples was processed by peak segmentation and curve fitting was performed. **Figure 7** shows the peak results; 0, 96, and 192 days in the figure represent the UV irradiation time. The types of C1s and O1s peaks and positions of binding energy and the proportion of each valence state of elements before and after UV irradiation are shown in the table.

As can be seen from **Table 2**, after UV irradiation, the C1s spectrum obtained three peaks through curve fitting, and the binding energies were 284.05, 285.75, and 287.45 eV, respectively. C4 content was low, and no fitting analysis was conducted for this kind of C atom. In terms of the changes in the relative contents of the three groups, C1 showed a downward trend, while C2 and C3 showed an upward trend. In the binding form of O-C-O, C=O, and O-C=O, the electron binding energy was larger, indicating that during the UV irradiation process, element C was constantly in contact with oxygen in the air, resulting in oxidative degradation. After the oxidation reaction, the chemical bonds on the surface of the *S. psammophila* sand barrier tend to be more stable, and the properties of more stable binding compounds are generated. However, with the increase of UV irradiation time, the change of this reaction is not linear; that is, a certain oxidation state is not proportional to the increase of UV irradiation time, during which a complex mutual conversion process occurs. It can be seen from the UV irradiation results that the oxidation states C2 and C3 of the *S. psammophila* sand barriers after UV irradiation show an increasing trend. After 96 days, C2 increases from 20.70% to 33.50%, and C3 increases from 6.29% to 10.52%. After 192 days, C2 increased to 38.97% and C3 increased to 10.85%. On the other hand, the non-oxygen group C1 decreased to 55.98% after 96 days and 50.18% after 192 days. 

**Table 2:** UV radiation aging process types and binding energy positions of C1s peaks.

Element constitution	Binding type	Binding energy (eV)	Time (days)		
			0	96	192
C1/%	C-C or C-H	284.05	73.01	55.98	50.18
C2/%	C-O	285.75	20.70	33.50	38.97
C3/%	O-C-O or C=O	287.45	6.29	10.52	10.85
C1/C2	——	——	3.53	1.67	1.29
C_ox_/C_unox_	——	——	0.37	0.79	0.99
O1/%	O-C=O	532.25	97.90	95.73	95.97
O2/%	C-O-, C=O, C-O-C, O-C=O	530.25	2.10	4.27	4.03
O/C	——	——	0.23	0.38	0.42

The oxidation degree of sand barrier carbon was calculated by C1s and expressed by C_ox_/C_unox_, according to previous studies (Stark and Matuana, 2004; Stark and Matuana, 2007).


$${\text{C}}_{\text{ox}}{\text{/C}}_{\text{unox}}{\text{= C}}_{\text{oxygenated}}{\text{/C}}_{\text{unoxygenated}}=\left(C2 + C3 + C4\right)/C1$$

**Figure 8:**
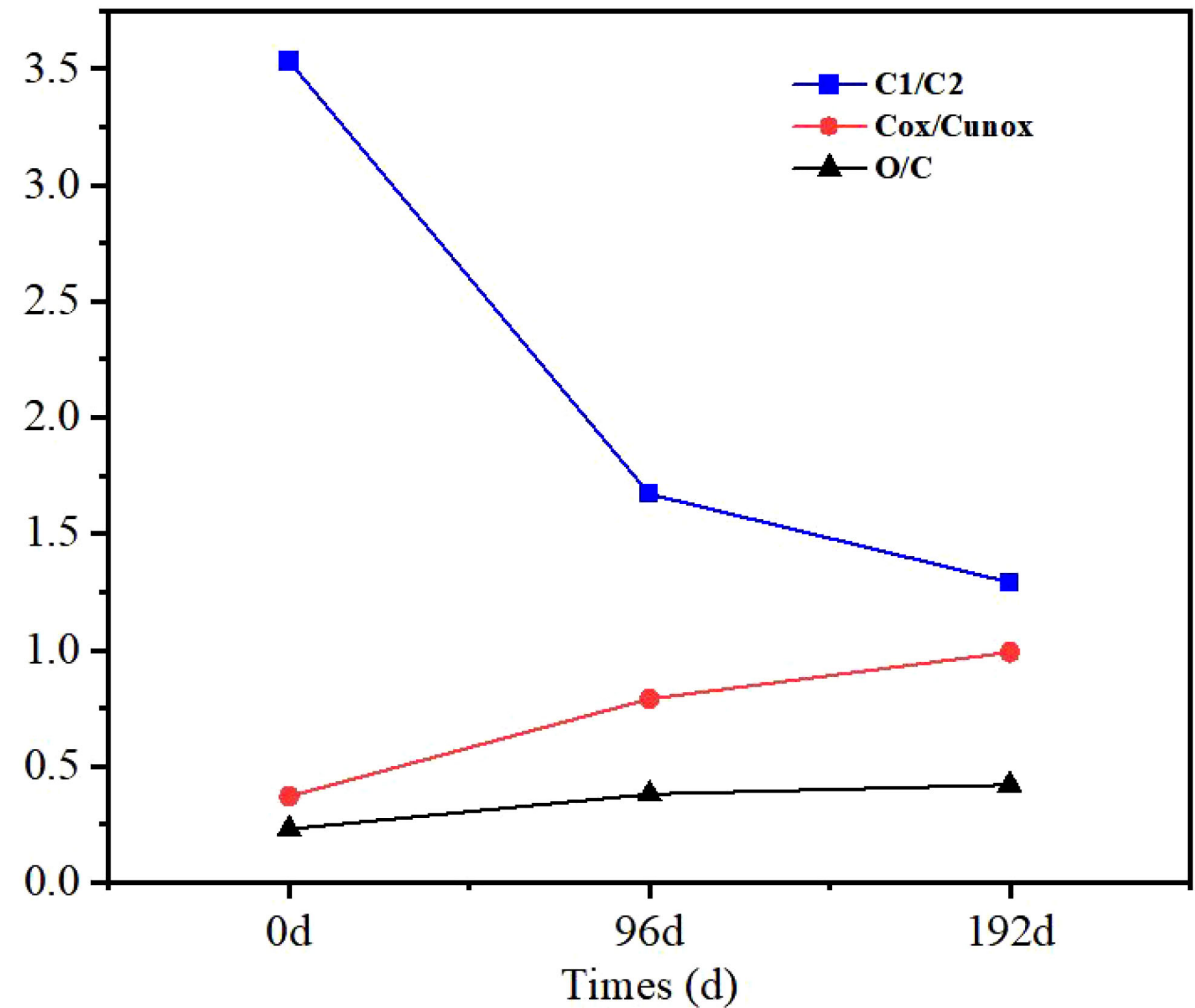
UV irradiation aging process and C1s and O/C changes of *S. psammophila* sand barriers.

The **Figure 8** shows the XPS changes of C1s, O/C, and O1s of sand barrier samples exposed to UV radiation at different times. The C1/C2 curve of the sand barrier shows that with the increase of UV radiation time, the ratio of C1 continues to decrease, and the UV radiation drops from 3.53 to approximately 1.67 after 96 days, with a more obvious decrease, and the UV radiation drops to 1.29 after 192 days. It can be seen from the C_ox_/C_unox_ curve that the contents of C2 and C3 show an increasing trend, and the UV radiation increases from 0.37 to 0.99 after 96 days and to 1.10 after 192 days, indicating that the quantities of hydroxyl, carbonyl, and carboxyl groups are increasing. O1 indicates that the binding form of O atom is C=O, O2 denotes that the binding form of O atom is C-O-, O1 mainly comes from the lignin of the *Salix* sand barrier, and O2 mainly comes from cellulose and hemicellulose. Before the sand barrier is exposed to UV radiation, O/C is 0.23, and C/O rises to 0.38 after 96 days of UV radiation and 0.42 after 192 days. In contrast, the O/C ratio showed a slow increasing trend, indicating that the relative content of lignin with low O/C may be decreasing, and again indicating that the lignin in the sand barrier was degraded after UV radiation and the lignin content was relatively reduced. This study can indirectly prove that lignin is less stable than polysaccharide under UV irradiation. In other words, the photostability of lignin is lower than that of polysaccharide, and it is the main component of photodegradation of the *Salix* sand barrier. It can be seen from the change of data that the chemical groups of *S. psammophila* sand barriers have a large amount of photooxidation reaction, which is one of the main reasons for the change of its performance.

## 4 Discussion

After eight cycles of UV irradiation treatment, a series of irreversible photoaging phenomena occurred, resulting in structural changes and alterations in the mechanical properties of *S. psammophila* sand barriers, thereby impacting the service life of the sand barriers. The UV irradiation commenced on day 96, and distinct variations were observed with increasing periods of UV exposure. The surface exhibited noticeable alterations in color and decreased lightness and glossiness, leaning toward yellowing and losing its original color. Both L and h values displayed similar changing trends. With the extension of UV irradiation time, it gradually decreases, while the red-green axis chromaticity index a*, yellow-blue axis chromaticity index b*, and color saturation C value gradually increase, and the brightness L and hue h value decrease at the same time, making the color of the sand barriers much darker with the UV irradiation treatment. After the sand barriers are exposed to UV irradiation, a good linear relationship between lightness and tone is seen. For the UV irradiation of the *S. psammophila* sand barriers, lightness can be used as a parameter to measure the color change. This conclusion is consistent with previous research results (Tolvaj, 2010; Tolvaj et al., 2011). Changes in the color of UV irradiation indicate that a photochemical reaction has begun to occur on the surface. Previous studies have shown that UV irradiation of natural wood materials is a free radical reaction, free radicals can be transferred freely in the surface layer, and free radicals can be generated after exposure to light, resulting in photochemical reactions (Hon, 2010). While natural wood materials belong to solid biological materials, the freedom of molecular movement will be limited to a certain extent, free radicals and oxygen required for photochemical reaction will diffuse from the surface to the plant body, and the reaction rate will slow down after reaching saturation state with the light reaction (Müller et al., 2003). As a natural wood material, *S. psammophila* sand barriers limit the transmission of free radicals, and with the progress of photochemical reaction, a protective layer will be formed on the surface, preventing the further development of UV irradiation. During the initial stage of UV irradiation, the surface layer of *S. psammophila* sand barriers undergoes rapid color changes. As the reaction progresses, limited diffusion conditions lead to a significant decrease in reaction speed, ultimately reaching a saturation state and slowing down the rate of color change. In addition to altering color, prolonged UV exposure also results in increased hardness and brittleness of the sand barrier material.

UV irradiation for 96 and 144 days is an important time change point to measure the damage degree of *S. psammophila* sand barriers; that is, when the sand barrier is subjected to the single factor of UV irradiation for 96 and 144 days, physical parameters, such as mass and basic density; mechanical properties, such as modulus of rupture and modulus of elasticity; and main chemical components of the sand barrier are lost to a certain extent. After 96 days of photoaging, the weight loss rate of *S. psammophila* sand barriers is approximately 2.84%, the basic density decreases by 4.09%, and the modulus of rupture decreases by 1.95%. The basic density decreased by 9.43% after 144 days. After 192 days of UV irradiation, the weight loss percentage decreased by 3.62%, the basic density decreased by 13.44%, the modulus of rupture decreased by 8.63, and the modulus of elasticity decreased by 6.74%. Our study further confirmed that with the increase of UV irradiation treatment time, the physical and mechanical properties of sand barriers were affected to some extent, and the degradation of cell wall materials led to the decrease of physical properties such as the mass, basic density, and dry shrinkage of *S. psammophila* sand barriers (Wang et al., 2022). The reason may be that after UV irradiation, UV rays are absorbed by polymers containing carbonyl groups and double bonds, and serious deterioration and chemical reactions occur in the interior, resulting in the break of macromolecular chains of wood materials, chemical structure changes, and material property deterioration. This conclusion is the same as the results of previous studies (Yu, 2015; Zhao, 2006). The results of each index indicate that the UV irradiation reaction is faster in the initial stage, representing a distinct chemical reaction process. This demonstrates that UV irradiation can alter the physical and mechanical properties of the sand barriers, albeit to a limited extent, and also highlights that UV irradiation of *S. psammophila* sand barriers is a prolonged process.

According to the SEM analysis after UV irradiation treatment, the degradation near the ray cells at the port of *S. psammophila* sand barriers is mainly caused by the difference in the structural size between the ray cells and the surrounding tissues (Hon et al., 1985), which is also one of the reasons for the change in the quality and main chemical composition of sand barriers. Both lignin and cellulose of *S. psammophila* sand barriers were degraded, and the fiber structure inside the sand barrier was destroyed. Since the lignin content in the intercellular layer was higher than that in the cell wall, the UV irradiation in sand barriers mainly occurred in the intercellular layer, which was consistent with previous research results (Kučera and Sell, 1987; Kuo and Hu, 2009). As can be seen from the results of the overall reduction of the main chemical components in the whole UV irradiation cycle, the lignin content, hemicellulose content, and cellulose content decreased by 23.12%, 14.30%, and 6.96%, respectively, after UV irradiation in the whole 0 to 192 days cycle. It shows that lignin is the main chemical component of UV irradiation, and only a small amount of cellulose is decomposed. In the FTIR analysis, the absorption peak of lignin at 1,610 cm^−1^ (C=C group of lignin skeleton), 1,510 cm^−1^ (vibration of lignin aromatic benzene ring skeleton), 1,460 cm^−1^ (in lignin and carbohydrates C-H), 1,425 cm^−1^ (C-H in lignin and hemicellulose), and 1,242 cm^−1^ (lignin C-O) was remarkably weakened after UV irradiation for 192 days, indicating that the lignin was degraded by UV irradiation and the lignin content was greatly reduced. This is similar to the conclusion of previous studies (Mitsui et al., 2001). UV irradiation-induced reduction in the content of the primary chemical constituents of sand barriers also results in diminished wood material properties (Garrote et al., 2001). The transition from light to dark color is primarily attributed to UV-induced degradation occurring predominantly in lignin, with alterations in lignin impacting the stability and durability of sand barriers to a certain extent. The nonlinear nature of the curve indicates that as UV irradiation time increases, the alteration in the properties of the sand barriers is not a simple physical or chemical change, but rather a more intricate process.

The XPS wide scanning spectrum and C1s peak analysis of *S. psammophila* sand barriers before and after UV irradiation showed that C1 showed a decreasing trend and C2 and C3 showed an increasing trend in terms of the relative contents of the three groups after 192 days of artificial UV irradiation. In the form of O-C-O, C=O, and O-C=O, the electron binding energy is larger, indicating that during the process of UV irradiation, the C element is constantly in contact with oxygen in the air, forming many kinds of C and O bonds. The presence of carbon atoms linked to a non-carbonyl oxygen atom, i.e., -C-O- bonds, forms chemical bonds akin to alcohols or ethers. This indicates the prevalence of carbon atoms in the cellulose and hemicellulose molecules of *S. psammophila* sand barriers, connected with the hydroxyl group -OH bond, specifically -O-C-O- or C=O. As the UV irradiation time increases, a significant change is observed in C3. This may be attributed to the presence of a C=O double bond in C3, which is relatively unstable compared to a C-O single bond and is susceptible to degradation under the influence of high-energy UV photons. Furthermore, prolonged UV irradiation leads to an increase in C2, initiating a new phase of degradation process. C2 represents a pure C-O single bond. Following the oxidation reaction, the chemical bonds on the surface tend to become more stable, leading to enhanced stability in bonding. Certain oxidation states do not directly increase under UV irradiation and exhibit nonlinear changes, indicating a complex mutual transformation process. Sun’s research revealed that wood materials are sensitive to UV irradiation, with significant changes occurring after 1 h of UV exposure; there was an increase in high binding energy ends and oxidation of carbon atoms on the wood surface. With the extension of UV irradiation time, the curve change of carbon valence states was nonlinear, not a simple physical and chemical change (Sun, 2011). C/O increased to 0.38 and 0.42 after 96 and 192 days of UV irradiation, respectively, and the O/C ratio showed a slow increasing trend, indicating that the relative content of lignin with low O/C was probably decreasing. This research finding can indirectly prove that lignin is less stable than polysaccharide under UV irradiation. Wang showed that polysaccharide components are less affected by UV irradiation (Wang et al., 2009). In essence, the light stability of lignin is lower than that of polysaccharides, making it the primary component affected by UV irradiation degradation in *S. psammophila* sand barriers. This finding aligns with the results of the Kalson lignin assay. The observed data changes indicate that an increase in the carbon-to-oxygen ratio, signifying oxidation of *S. psammophila* sand barriers, is a key factor influencing its performance. This suggests that light primarily contributes to the degradation of lignin in *S. psammophila* sand barriers and underscores that UV-induced degradation of these barriers is a protracted process.

## 5 Conclusion

(1) UV irradiation for 96 and 144 days serves as crucial time points to measure the UV degradation degree of *S. psammophila* sand barriers. After UV irradiation, the physical properties of sand barriers, mainly mass and basic density, and the mechanical properties, mainly modulus of rupture and modulus of elasticity, are lost to a certain extent. After 192 days of UV irradiation, the mass loss percentage decreased by 3.62%, and the MOR and MOE decreased by 8.63 and 6.74%, respectively.

(2) As the duration of UV irradiation increases, there is a discernible decrease in both lightness and hue, leading to an expansion in color difference and a darkening of the overall color. A strong linear correlation exists between changes in lightness and hue, suggesting that lightness can serve as a reliable metric for assessing color variations induced by UV irradiation on *S. psammophila* sand barriers.

(3) After 192 days of UV irradiation, the lignin, hemicellulose, and cellulose content in *S. psammophila* sand barriers decreased by 23.12%, 14.30%, and 6.96%, respectively. The carbon binding form underwent changes with a noticeable decrease in C1 content and an increase in C2 and C3 content, leading to gradual increases in the oxidation state and binding energy of carbon. The absorption peak intensity of the lignin aromatic ring structure at 1,610, 1,510, 1,460, 1,425, and 1,242 cm^−1^ significantly weakened, indicating the occurrence of photooxidation reaction. Polysaccharides (cellulose and hemicellulose) in *S. psammophila* sand barriers were less affected by UV irradiation.

(4) The lignin in *S. psammophila* sand barriers is the primary factor contributing to UV degradation and discoloration. Therefore, it is essential to manage the degradation behavior of the sand barrier by inhibiting lignin reactions.

## Data Availability

The original contributions presented in the study are included in the article/supplementary material. Further inquiries can be directed to the corresponding author.
